# Speech intelligibility and talker gender classification with noise-vocoded and tone-vocoded speech

**DOI:** 10.1121/10.0006285

**Published:** 2021-09-20

**Authors:** Sarah Villard, Gerald Kidd

**Affiliations:** Department of Speech, Language and Hearing Sciences & Hearing Research Center, Boston University, 635 Commonwealth Avenue, Boston, Massachusetts 02215, USAsvillard@bu.edu, gkidd@bu.edu

## Abstract

Vocoded speech provides less spectral information than natural, unprocessed speech, negatively affecting listener performance on speech intelligibility and talker gender classification tasks. In this study, young normal-hearing participants listened to noise-vocoded and tone-vocoded (i.e., sinewave-vocoded) sentences containing 1, 2, 4, 8, 16, or 32 channels, as well as non-vocoded sentences, and reported the words heard as well as the gender of the talker. Overall, performance was significantly better with tone-vocoded than noise-vocoded speech for both tasks. Within the talker gender classification task, biases in performance were observed for lower numbers of channels, especially when using the noise carrier.

## 1. Introduction

Since the development of the vocoder in the first half of the 20th century (Dudley, 1939), many scientific pursuits and clinical applications have arisen that were based on the underlying technique; that is, imposing the amplitude envelopes derived on a band-by-band basis from a broadband signal (e.g., speech or music) on a specified carrier (e.g., continuous noise, tones, or pulse trains). The earliest work focused on the scientific questions surrounding the information that must be retained in the vocoded signal to preserve its essential properties and to use that information to synthesize accurate duplicates of the original signal. Although there is a wide range of applications of vocoding in modern communications and music synthesis technology, the most common topic areas in the speech perception and psychological acoustics fields involve using vocoding to control various aspects of a signal in the study of normal auditory processes (e.g., pitch, binaural hearing, masking) and as a useful (although limited) analog to the information conveyed by cochlear implants (CIs), especially with regard to speech recognition (Shannon *et al.*, 1995).

In the current study, we compared the perception of one “essential property” of human voice—perceived gender—with speech intelligibility for two common types of carriers: broadband noise (i.e., noise vocoder) and multiple widely spaced pure tones (i.e., tone vocoder). Our ultimate goal for this line of work is to obtain a better understanding of how perceived talker gender can be exploited in multisource “cocktail party” situations (cf. recent series of reviews in Middlebrooks *et al.*, 2017) where the listener must focus or redirect auditory attention in highly uncertain listening situations. Recent work has established that differences in talker gender can be a powerful cue to overcoming masking in multiple-talker listening situations (e.g., Kidd *et al.*, 2016) and that the control of the stimulus parameters that is possible *via* vocoding can be useful in determining the different factors that contribute to speech-on-speech masking (e.g., Arbogast *et al.*, 2002; Swaminathan *et al.*, 2016). Furthermore, although it is not our intent to draw conclusions about the perceptual abilities of CI listeners by using vocoder simulations in normal-hearing (NH) listeners, we do hope to establish a baseline against which future studies with CI listeners may be compared, in keeping with many past studies that have contributed to this body of knowledge (e.g., Fu *et al.*, 2004; Whitmal *et al.*, 2007; Yun *et al.*, 2021).

With respect to the rationale for comparing performance for noise- and tone-vocoders, numerous previous studies have shown that both noise vocoding (e.g., Dorman *et al.*, 1997; Scott *et al.*, 2006; Shannon *et al.*, 1995; Whitmal *et al.*, 2007; Yun *et al.*, 2021) and tone vocoding (e.g., Dorman *et al.*, 1997; Loizou *et al.*, 1999; Whitmal *et al.*, 2007) negatively affect speech intelligibility, with decreasing numbers of vocoded channels generally resulting in poorer performance. However, the two types of carriers are acoustically and qualitatively quite different. The random fluctuations inherent to noise add variability to the within-channel envelopes of the noise-vocoded stimuli. Additionally, with noise vocoding, there typically is a substantial overlap between adjacent channels, often creating a stimulus with a relatively continuous spectrum. In contrast, with tone vocoding, the stimulus comprises a series of discrete spectral peaks with relatively little spectral overlap. Furthermore, the side bands created by modulated tone carriers may be perceptually salient and could influence task performance; cf. Schvartz and Chatterjee, 2012). As shown by Arbogast *et al.* (2002, 2005) the very narrow spectral representations of intelligible speech that are possible with tone vocoding are well-suited for studying masking and comparing masking in listeners with normal hearing and with hearing loss.

As with performance on speech intelligibility tasks, performance on talker gender classification tasks also is negatively affected when speech is noise- or tone-vocoded, particularly at lower numbers of channels (e.g., Fu *et al.*, 2004; Gonzalez and Oliver, 2005; Schvartz and Chatterjee, 2012). The ability of listeners to reliably perceive talker gender can have important implications for speech intelligibility, particularly under speech-on-speech masking conditions. Previous studies have provided clear evidence that gender differences between target and masker talkers typically result in a substantial release from masking, allowing listeners to successfully understand target speech at significantly lower target-to-masker ratios (e.g., Brungart, 2001; Kidd *et al.*, 2016; Kidd *et al.*, 2019). Any alteration/distortion of the speech signal that renders it more difficult for a listener to distinguish talker gender deprives the listener of that powerful source segregation cue and could diminish the typical release from masking that would otherwise occur. Indeed, one study directly examining the effect of vocoding on the release from masking provided by different-gender talkers found that the benefit disappeared when speech was noise- or tone-vocoded into 15 channels (Brungart *et al.*, 2006). Furthermore, the degree of masking release provided by talker gender cues has also been examined in CI users, with the results indicating that these listeners can use acoustic cues related to talker gender to some extent (Willis *et al.*, 2021), but cannot categorize gender as well as non-CI listeners (e.g., Fu *et al.*, 2005; Fuller *et al.*, 2014).

Despite the loss of spectral information that results from vocoding, listeners (in particular, younger listeners; e.g., Schvartz and Chatterjee, 2012) are typically able to achieve a high level of performance on both speech intelligibility tasks and gender classification tasks with both noise- and tone-vocoded speech, even when the signal consists of relatively few channels. This high performance is thought to be due largely to the use of the temporal envelope cues that remain available in the reduced number of frequency regions (Dorman *et al.*, 1997; Roberts *et al.*, 2011; Shannon *et al.*, 1995; Souza and Rosen, 2009). Listeners may also be able to boost performance by drawing on linguistic knowledge, as well as on knowledge about the probable speech content (particularly in experimental paradigms involving closed-set stimuli). Good performance on talker gender classification tasks using vocoded stimuli is thought to be achieved through temporal envelope periodicity cues that provide information about fundamental frequency (Schvartz and Chatterjee, 2012), as well as vocal tract length cues that provide information about formants (Fuller *et al.*, 2014).

The reported minimum numbers of vocoded channels needed to achieve high levels of performance on these two types of tasks vary somewhat from study to study; however, these variations are likely attributable to differences in experimental parameters such as the type of stimuli used (e.g., sentences vs vowel tokens) and/or the envelope cutoff frequency applied. One study investigating the effect of noise vocoding on speech intelligibility observed high performance (80% or more correct) as long as the signal was divided into 8 or more noise-vocoded channels (Scott *et al.*, 2006), while another study reported high performance with as few as three noise-vocoded channels (Shannon *et al.*, 1995). With tone-vocoded stimuli, listeners have been observed to achieve high performance on a speech intelligibility task with as few as five channels (Loizou *et al.*, 1999). Regarding talker gender classification tasks, studies have reported that with noise-vocoded speech, listeners are able to achieve performance levels over 80% correct with as few as eight channels (Schvartz and Chatterjee, 2012), six channels (Gonzalez and Oliver, 2005), or even four channels (Fu *et al.*, 2004); the number of channels required to achieve this performance level may be dependent on the type of speech stimuli and vocoding parameters used. While there is relatively little existing research on talker gender classification with tone-vocoded speech, one study found that listeners could reliably classify talker gender when tone-vocoded speech consisted of only three channels (Gonzalez and Oliver, 2005).

Collectively, these previous studies suggest that high performance on both speech intelligibility and talker gender classification tasks may be achieved when speech is either noise- or tone-vocoded. However, there have been relatively few direct comparisons (i.e., in the same group of subjects) of listener performance with noise-vocoded vs tone-vocoded speech on these two tasks, and *post hoc* comparisons between different studies are not always possible due to differences in the types of stimuli and vocoding parameters used. Several previous studies have compared speech intelligibility for noise- vs tone-vocoded speech (e.g., Dorman *et al.*, 1997; Whitmal *et al.*, 2007; Xu *et al.*, 2021). Results of these studies were somewhat mixed, with some evidence suggesting an advantage of tone vocoding over noise vocoding (Dorman *et al.*, 1997; Whitmal *et al.*, 2007) but other evidence indicating similar performance between the two (Xu *et al.*, 2021). The only previous study (to our knowledge) that has compared talker gender classification with noise-vocoded vs tone-vocoded speech found better performance with tone vocoding; however, this study used two different envelope cutoffs for the two types of vocoding, resulting in a slightly less direct comparison (Gonzalez and Oliver, 2005).

The questions addressed by the current study, which were evaluated for noise-vocoded as well as for tone-vocoded speech, are:

(1) At what point does the degradation of the speech stimulus due to decreasing numbers of frequency channels compromise the ability to distinguish the gender of a talker (and, presumably, to use gender differences as a basis for talker segregation)?
(2) At that point of degradation, how intelligible is the speech?

Because performance may vary significantly across individuals, our approach was to measure performance on all tasks using the same subjects. Because of the inherent acoustic and perceptual differences between tone- and noise-vocoded speech stimuli, we sought to assess performance using both types of speech stimuli. Our expectation, based on previous work, was that listeners would require slightly fewer tone-vocoded channels than noise-vocoded channels to achieve similar levels of performance, on both tasks. This hypothesis was based on the small advantage of tone vocoding seen in some previous work on speech intelligibility, as well as the knowledge that amplitude modulation of tone-vocoded speech creates side bands, which may provide additional spectral cues that could aid in talker gender classification and that are not available in noise-vocoded speech (cf. Gonzalez and Oliver, 2005; Schvartz and Chatterjee, 2012). Because speech intelligibility performance can vary based on the precise stimuli and vocoding parameters used, we had no specific prediction about the number of channels that would be required to achieve a high level of performance (e.g., 80% correct) but expected that performance would decrease substantially as the number of channels dropped below eight.

## 2. Methods

### 2.1 Participants

Eight participants (aged 19–23; three male, five female), with NH in both ears as indicated by a pure-tone hearing screening, were recruited as listeners in this experiment. Participants had limited if any experience with vocoded speech. They were compensated for their time on an hourly basis.

### 2.2 Stimuli

Stimuli consisted of pre-recorded single words spoken by 16 talkers: eight male (mean *F*0 = 124 Hz, range = 105–153 Hz) and eight female (mean *F*0 = 196 Hz, range = 173–234 Hz). All words were drawn from a matrix-style corpus that included 40 one-syllable words: eight names, eight verbs, eight numbers, eight adjectives, and eight nouns (see Table 1). On each trial, a word was randomly drawn from each of these categories, and the five words were concatenated into a grammatical sentence with the structure <name> <verb> <number> <adjective> <noun>, e.g., “Bob saw three red shoes.” Each sentence was spoken by a single talker. All types of sentences, including natural sentences, were band-passed from 80 to 8000 Hz. Sentences were presented either with no further processing (“natural speech”), noise-vocoded, or tone-vocoded. Each noise- or tone-vocoded sentence contained 1, 2, 4, 8, 16, or 32 channels; this created a total of 13 experimental conditions including the natural speech condition.

**TABLE 1.** Experimental corpus.

Bob	bought	2	big	bags
Gene	found	3	cheap	cards
Jane	gave	4	green	gloves
Jill	held	5	hot	hats
Lynn	lost	6	new	pens
Mike	saw	8	old	shoes
Pat	sold	9	red	socks
Sue	took	10	small	toys

### 2.3 Vocoding

For both noise vocoding and tone vocoding, the total band-passed frequency range (80–8000 Hz) was subdivided into the designated number of channels for a given condition, with bandwidths and center frequencies chosen according to the algorithm used by Swaminathan *et al.* (2016) (see also Smith *et al.*, 2002). Within each channel, a Hilbert transform was applied to the speech waveform and the amplitude envelope function was obtained (the phase function was discarded). Each within-channel envelope function was low-pass filtered at 400 Hz and multiplied in the time domain by the appropriate carrier for that channel (either noise or pure-tone) and the outputs of the different bands were summed to create the processed speech signal. For noise vocoding, a different sample of a broadband noise carrier was generated for each trial. For tone vocoding, a center frequency—the arithmetic center between the upper and lower band cutoffs—was identified for each designated band, and a sinewave was generated at that frequency (starting phase chosen randomly between 0 and π radians on each trial). Please see Table 2 for the center frequencies and bandwidths of the channels in each condition.

**TABLE 2.** Center frequencies and bandwidths for each of the numbers of channels used in the experiment. Note: cf, center frequency; bw, bandwidth, both in Hz. Each bandwidth is listed directly below its corresponding center frequency.

*1*	**cf**	**4040**							
	*bw*	*7920*							
*2*	**cf**	**626**	**4586**						
	*bw*	*1092*	*6828*						
*4*	**cf**	**236**	**782**	**2147**	**5561**				
	*bw*	*312*	*780*	*1951*	*4877*				
*8*	**cf**	**140**	**296**	**543**	**933**	**1550**	**2525**	**4067**	**6506**
	*bw*	*121*	*191*	*302*	*478*	*756*	*1195*	*1890*	*2988*
*16*	**cf**	**107**	**167**	**243**	**339**	**459**	**610**	**800**	**1039**
	*bw*	*54*	*67*	*85*	*106*	*134*	*168*	*212*	*266*
	**cf**	**1339**	**1717**	**2192**	**2790**	**3541**	**4486**	**5674**	**7168**
	*bw*	*335*	*421*	*529*	*666*	*837*	*1053*	*1324*	*1664*
*32*	**cf**	**93**	**119**	**149**	**183**	**221**	**263**	**311**	**364**
	*bw*	*25*	*28*	*32*	*36*	*40*	*45*	*50*	*56*
	**cf**	**424**	**490**	**566**	**650**	**744**	**850**	**969**	**1102**
	*bw*	*63*	*71*	*79*	*89*	*100*	*112*	*125*	*141*
	**cf**	**1251**	**1418**	**1606**	**1816**	**2052**	**2317**	**2614**	**2947**
	*bw*	*158*	*177*	*198*	*223*	*250*	*280*	*314*	*352*
	**cf**	**3320**	**3738**	**4208**	**4734**	**5324**	**5986**	**6728**	**7560**
	*bw*	*395*	*442*	*496*	*556*	*624*	*700*	*785*	*880*

### 2.4 Procedure

Participants were seated in a double-walled sound booth in front of a computer monitor. Stimuli were presented diotically *via* headphones. For all tasks, participants used a mouse click to select responses from a graphical user interface.

Prior to the experiment, participants listened to a series of sample sentences, two in each condition (one spoken by a male talker and one spoken by a female talker), without providing any response, to familiarize themselves with the vocoded stimuli used in the experiment. Next, an instructional script was read aloud to participants, explaining that in some experimental blocks they would be asked to report the words that they had heard, and in other blocks, they would be asked to report whether a talker sounded male or female. This script included a statement explicitly recognizing that while gender is a complex construct encompassing more than the binary categories male and female, in this experiment participants would be asked to choose between those two response options. It should be noted that while the terms “female” and “male” may often be used to refer to biological sex (rather than to gender), in the current study we use these terms to denote perception of gender on the part of the listener, in keeping with the terminology used in current literature on listener perception of talker gender (e.g., Brown *et al.*, 2021). The script also communicated to participants that throughout the experiment they would hear an equal number of sentences spoken by male and female talkers.

Each participant completed 8 experimental blocks, four of which were speech intelligibility blocks and four of which were talker gender classification blocks. Block order was counterbalanced across participants. All stimuli were presented at 60 dB sound pressure level (SPL). For speech intelligibility blocks, participants reported each of the five words one by one, in the order heard, by selecting each from a list of eight options. For gender classification blocks, participants were presented with the two-alternative forced choice of “male” or “female.” In total, across all blocks, each participant heard 24 sentences in each condition, half of which were spoken by a male talker and half of which were spoken by a female talker. The specific male or female talker was randomly selected for each trial. No feedback on performance was provided at any point during the experiment. Participants were encouraged to take breaks between blocks as needed.

## 3. Results

**FIG. 1:**
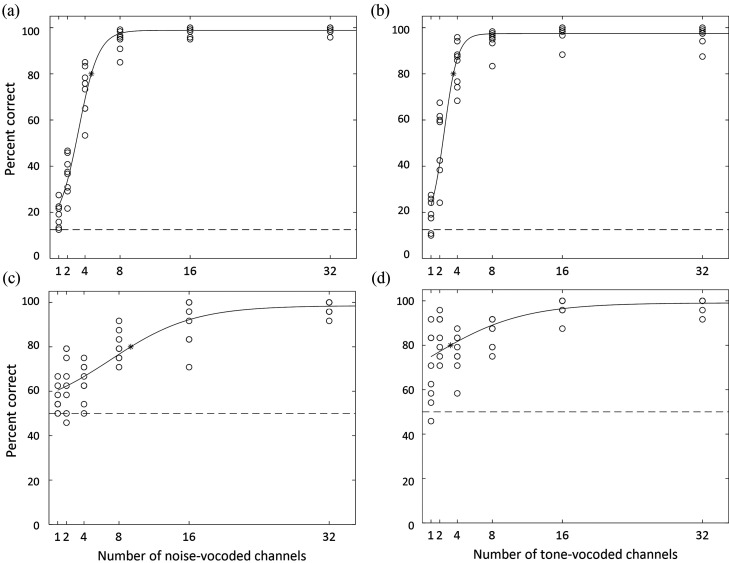
Psychometric functions estimated for (a) word identification of noise-vocoded speech (slope, 0.220). (b) Word identification of tone-vocoded speech (slope, 0.311). (c) Talker gender classification of noise-vocoded speech (slope, 0.057). (d) Talker gender classification of tone-vocoded speech (slope, 0.047). Open circles indicate performance levels for individual participants, while asterisks indicate the number of channels, based on the calculated function, that would be necessary for 80% correct performance. Dotted lines indicate chance-level performance.

A logistic psychometric function estimating the relationship between the number of channels and percent correct was calculated for each participant based on the obtained data set, for each task, in the noise-vocoded as well as in the tone-vocoded condition; the parameters of these functions were then averaged to create a combined function for each task in each vocoded condition (see Figs. 1(a)–1(d)]. Note that because the talker gender identification task was a two-alternative forced choice task (unlike the speech intelligibility task, which offered eight alternatives for each word), the chance of guessing correctly for this task was higher (50% correct), and therefore its two functions intersect the ordinate at a higher point. From each of the four functions, the estimated number of channels required to achieve 80% correct on each task was identified. On the speech intelligibility task, 80% correct performance corresponded to 4.7 channels for noise-vocoded speech and 3.6 channels for tone-vocoded speech. On the talker gender identification task, 80% correct performance corresponded to 9.3 channels for noise-vocoded speech and 3.2 channels for tone-vocoded speech.

The locations of these 80% correct points, along with overall visual inspection of the data presented in Figs. 1(a)–1(d), suggested that high performance on a given task was achieved with fewer channels when speech was tone-vocoded than when speech was noise-vocoded. Two repeated-measures analyses of variance (RM-ANOVAs) were performed, one for each task, to directly examine the effect of type of vocoding as well as number of channels. Because listener performance began to approach ceiling at higher numbers of channels for both tasks and both types of vocoding, an arcsine transform (Studebaker *et al.*, 1987) was first performed on the entire data set of each participant's proportion correct on each condition (a total of 104 data points). The data points included in the following ANOVAs were taken from the output of the arcsine transform. A 2 × 6 RM-ANOVA examining the effect of vocoding type (speech, noise) and number of channels (1, 2, 4, 8, 16, 32) on performance for the word identification task revealed a significant effect of vocoding type, *F*(1, 7) = 7.33, *p* < 0.05, as well as a significant effect of number of channels, *F*(5, 35) = 315.90, *p* < 0.001 and a significant interaction effect, *F*(5, 35) = 15.99, *p* < 0.001. A similar 2 × 6 RM-ANOVA examining the effect of vocoding type and number of channels on performance for the talker gender classification task revealed a significant effect of vocoding type, *F*(1, 7) = 16.75, *p* < 0.01 and a significant effect of number of channels, *F*(5, 35) = 64.31, *p* < 0.001 but no significant interaction effect. These analyses confirmed that, in addition to expected effects of number of channels, performance was significantly better with tone-vocoded than with noise-vocoded speech on both tasks.

**FIG. 2.**
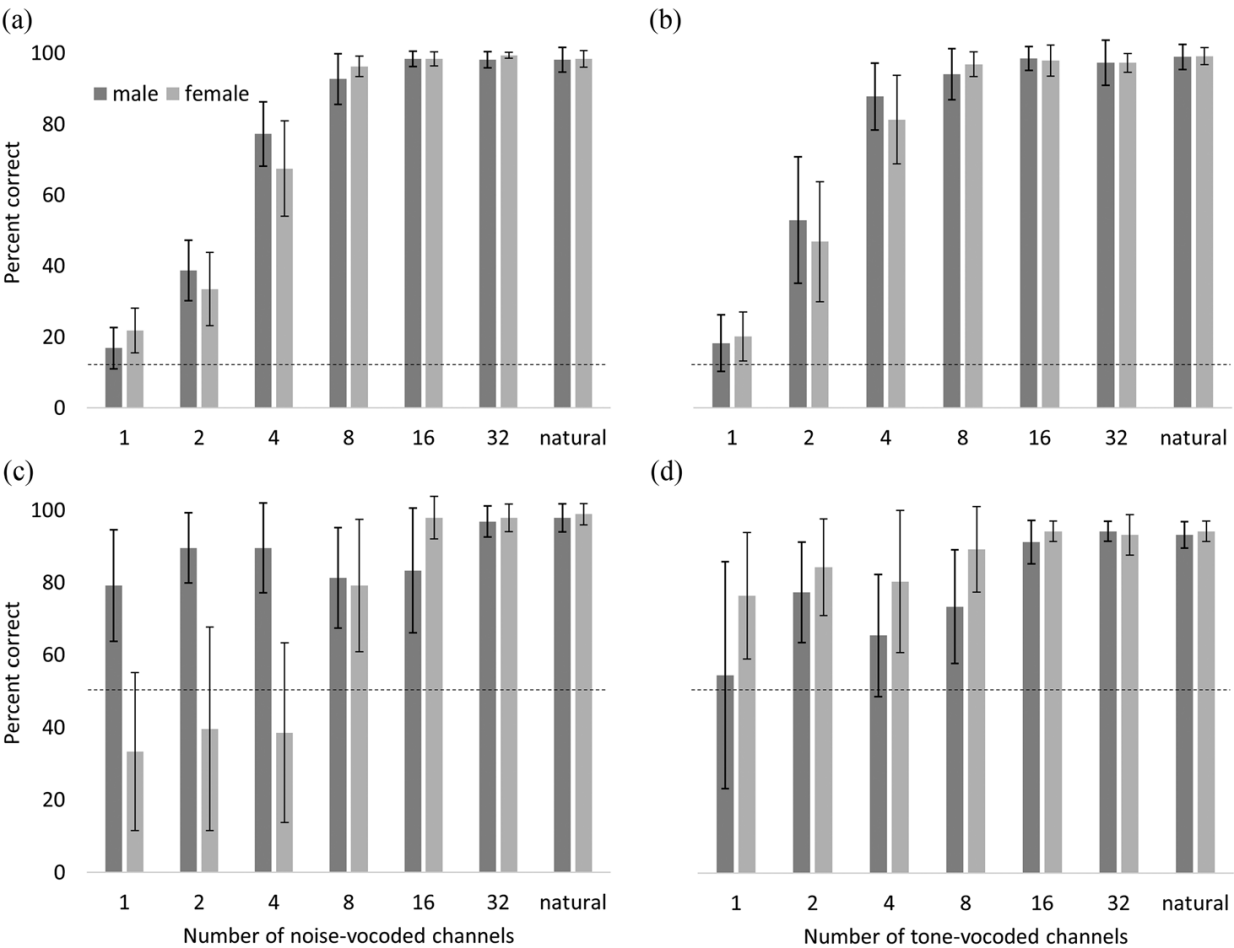
(a) Percent correct word identification by number of noise-vocoded channels, as compared to natural speech (last entry on abscissa in each panel). (b) Percent correct word identification by number of tone-vocoded channels, as compared to natural speech. (c) Percent correct talker gender classification by number of noise-vocoded channels, as compared to natural speech. (d) Percent correct talker gender classification by number of tone-vocoded channels, as compared to natural speech. Error bars indicate standard deviations across participants. Dotted lines indicate chance-level performance.

To further investigate factors that may have helped drive performance, performance on trials where sentences were spoken by male talkers was compared to performance on trials where sentences were spoken by female talkers [see Figs. 2(a)–2(d)]. Initial visual inspection of these results suggested that for the word identification task, performance was similar regardless of the actual gender of the talker. However, for the talker gender classification task, an apparent bias was observed, such that at lower numbers of channels (specifically 1, 2, and 4 channels), listeners were disproportionately likely to report that a noise-vocoded sentence had been spoken by a male talker. Note the performance below the 50% chance point in Fig. 2(c) is a consequence of this bias. A possible complementary bias was also observed for tone-vocoded sentences at lower numbers of channels (1, 2, 4, and possibly 8), where listeners appeared more likely to report that the talker had been female. However, notable listener-to-listener variability was also observed at these lower numbers of channels.

**FIG. 3.**
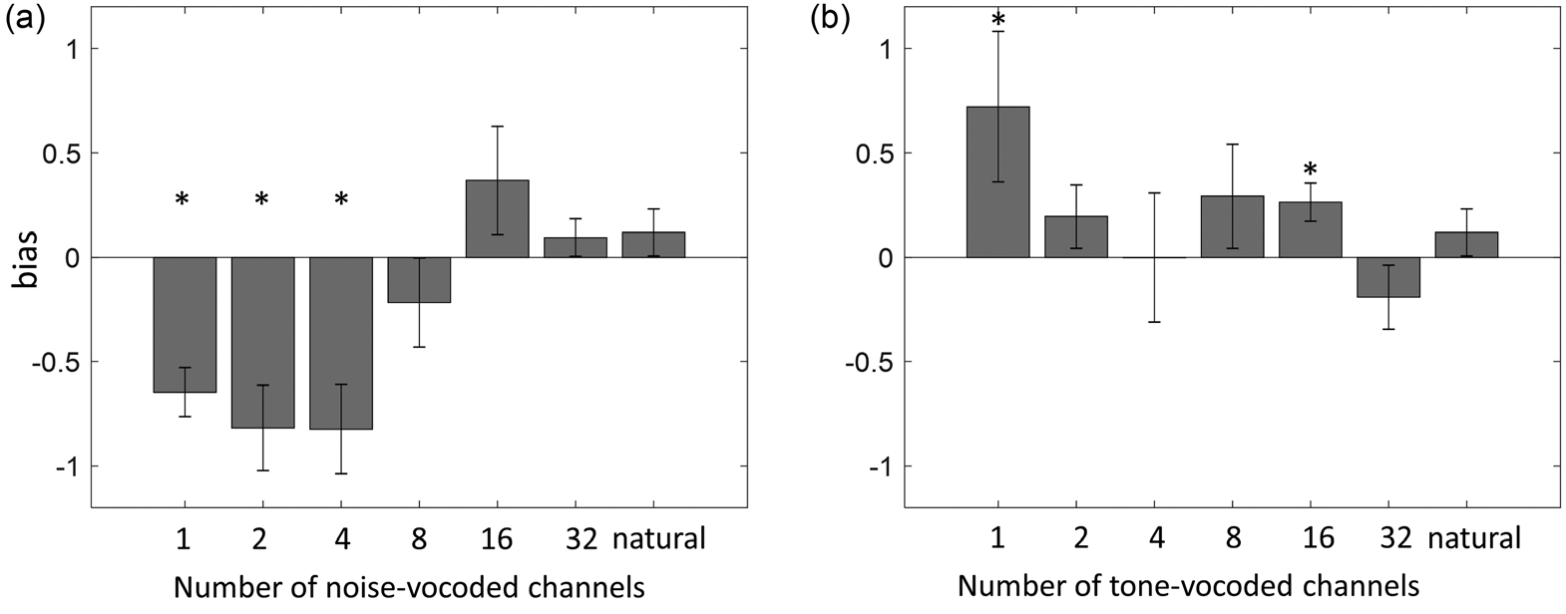
(a) Bias by number of noise-vocoded channels, as compared to natural speech, in the talker gender identification task. (b) Bias by number of tone-vocoded channels, as compared to natural speech, in the talker gender identification task. Asterisks indicate conditions where bias was significantly different from zero following a correction for multiple comparisons. Error bars indicate standard deviations across participants.

To further examine the apparent biases in performance for the talker gender identification task noted during visual inspection, an analysis of response bias was undertaken for each of the 13 conditions. First, within a given condition, hits, misses, false alarms, and correct rejections for all tokens where the correct response was “male” were counted with a value of 0.5 added to each count to avoid any counts of zeros. Next, the proportion of hits and proportion of false alarms were calculated. Finally, bias was calculated in matlab (The MathWorks, Natick, MA), using the following syntax: bias = −0.5 * [norminv (propHits) + norminv (propFAs)]. Bias for each condition is shown in Figs. 3(a) and 3(b). To determine for which conditions the amount of bias differed significantly from zero, 13 one-sample t-tests were conducted, one for each condition. The alpha level was adjusted for multiple comparisons by dividing 0.05 by the number of comparisons (13), yielding an adjusted alpha of 0.0038. The conditions for which bias remained significant following this adjustment were: 1-channel noise vocoding (*p* < 0.001), 2-channel noise vocoding (*p* < 0.001), 4-channel noise vocoding (*p* < 0.001), 1-channel tone vocoding (*p* < 0.001), and 16-channel tone vocoding (*p* < 0.001).

## 4. Discussion

This study examined listener performance on a word identification task and a talker gender classification task. The speech was either unprocessed or was noise-vocoded or tone-vocoded using various numbers of spectral channels. In general, the number of channels required for listeners to reliably identify words and classify talker gender as male or female in the current study was similar to the number of channels reported in previous studies. However, because the current study used the same stimuli and vocoding parameters throughout, direct comparisons were possible between performance with noise-vocoded and tone-vocoded speech for these two tasks.

One general observation is that, as predicted, performance on both tasks was shown to be significantly better with tone-vocoded than with noise-vocoded speech for low numbers of channels where performance was well below ceiling. This trend is consistent with several previous studies that have investigated related questions pertaining to noise-vocoded and tone-vocoded speech. For example, Whitmal *et al.* (2007) examined listener performance on a speech intelligibility task in which the stimuli were processed into six noise-vocoded or six tone-vocoded channels and were presented both in quiet and with noise or speech babble maskers. They found better speech intelligibility with tone-vocoded stimuli in both the masked and unmasked listening conditions when stimuli consisted of sentences (though when stimuli consisted of consonants alone, the advantage of the tone carrier was observed only in the masked listening condition). Dorman *et al.* (1997) also observed a small advantage of tone vocoding under certain conditions (though not when sentences were used as stimuli). Not all studies, however, have found a noticeable advantage of tone vocoding in speech intelligibility tasks (e.g., Xu *et al.*, 2021). These mixed findings could be due to differences in methodology, vocoding parameters, or features of the stimuli.

Previous work on gender classification of noise-vocoded vs tone-vocoded speech has yielded similar results. Gonzalez and Oliver (2005) found that listeners required fewer channels to achieve high talker gender classification performance with tone-vocoded than with noise-vocoded speech. While this contrast may have been affected by the different envelope cutoffs used for the two types of vocoding (160 Hz cutoff for noise-vocoded vs 400 Hz cutoff for tone-vocoded), the authors still concluded that fewer channels are needed for accurate talker gender classification with tone-vocoded than noise-vocoded speech, an interpretation that is consistent with the results of the current study and may be due to the side bands produced by modulation of tone-vocoded speech (Schvartz and Chatterjee, 2012).

It is also of interest to compare performance on the two tasks with respect to the fidelity of the stimulus as determined by the number of vocoded channels applied. For the speech identification task, performance rapidly improved with the number of channels available for both noise and tone carriers. For both types of vocoding, the slopes of the psychometric functions were steeper for the word identification task than for the gender classification task (see Fig. 1), resulting in asymptotic performance on speech identification clearly achieved by eight channels. In contrast, reaching asymptote on gender classification required 16 channels. Thus, there was a fairly wide range of stimulus resolution over which intelligibility was high, yet extraction of the features signaling gender was reduced. The extent to which this finding—better speech intelligibility than gender classification for small numbers of channels—generalizes to open set speech or to other methods of measuring performance on these tasks currently is not known. It is also not known whether an analogous trend would be present for CI subjects with respect to the number of functional channels. Further work is required to make these determinations.

Another notable (albeit incidental) finding of the current study consisted of significant bias exhibited by participants towards classifying noise-vocoded speech as being spoken by a male talker in the 1-, 2-, and 4-channel conditions. A significant bias towards classifying tone vocoded speech as being spoken by a female talker was also observed in the 1-channel condition, as well as in the 16-channel condition (though it should be noted that this last result was likely driven primarily by low subject-to-subject variability due to a ceiling effect in the 16-channel tone vocoded condition). In order to rule out the possibility that the biases seen in the talker gender classification task might have been driven by misclassification of specific talkers, we compared performance on this task across individual talkers. Only one talker (a male) stood out from the others as being frequently misclassified as female, while the remaining talkers appeared to be misclassified at roughly equivalent rates. We therefore concluded that these biases did not stem from specific talkers but rather were systematic across talkers.

The reasons for these biases are still unclear; however, some possible explanations are listed here. Certainly, a possible reason for the bias in favor of classification of tone-vocoded speech talkers as female in the 1-channel vocoded condition could be that the center frequency for this condition was 4040 Hz. When asked to classify according to gender, listeners may perceptually “search” the available acoustic information for a fundamental frequency, and, finding none, may focus on the center frequencies of the vocoded channels instead. While 4040 Hz is much higher than any fundamental frequency present in natural speech for a talker of any gender, listeners may simply have judged that it was closer to the fundamental frequency of a female than a male voice. This explanation, if correct, might also be expected to hold true for the 2-channel and 4-channel tone-vocoded conditions; however, the bias seen with these numbers of channels was not significant, either because the relatively low sample size of the study did not provide enough statistical power for these biases to reach significance or because the additional spectral information available in the 2-channel and 4-channel conditions reduced the presence of bias in some way.

It is more difficult to account for the more pronounced bias towards identifying the talker as male when speech with lower numbers of noise-vocoded channels was presented. Although this bias has not, to our knowledge, been previously discussed in the literature on vocoded speech, results published by Schvartz and Chatterjee (2012) on the ability of young NH listeners to classify talker gender with noise-vocoded speech containing very low numbers of channels and the same envelope cutoff frequency used in the current study (400 Hz) do appear to show somewhat higher overall performance with talkers whose fundamental frequencies (prior to vocoding) would be typically considered male (100–175 Hz) than with talkers whose fundamental frequencies would typically be considered female (200–275 Hz). Under these conditions, listener performance was highest for male talkers with especially low fundamental frequencies. However, it is not clear whether the difference between male and female talkers reached significance in that study. Regardless, the factors underlying the observed bias in the current study to classify talkers as male when speech was noise-vocoded with fewer than eight channels is difficult to account for. It is possible that noise-vocoding speech may produce or amplify acoustic characteristics that listeners perceive as being associated with male voices, and/or may remove or reduce acoustic characteristics that listeners perceive as being associated with female voices. Further research is needed to confirm and account for this bias. It is also notable that there was substantial variability in performance across listeners on this task, particularly at the more challenging lower numbers of channels.

In summary, the results from the current study suggest that when presented with a vocoded speech signal, listeners require fewer channels to understand the words in the signal and identify the gender of the talker when tone vocoding vs noise vocoding is used. Furthermore, asymptotic performance for speech recognition was evident for fewer channels than for gender classification for both noise and tone vocoding. The results indicate that in future experiments requiring high performance on both types of tasks, noise-vocoded speech should have a minimum of eight vocoded channels and tone-vocoded speech should have a minimum of four channels (given sentence-level stimuli and a high envelope cutoff frequency as used in the current study). Additionally, analysis of the results suggests the existence of listener biases in classifying vocoded speech as male or female, depending on the type of vocoding used. Because of the small sample size of the study, the results discussed here should be considered preliminary and invite further investigation using a larger number of participants.

These findings, particularly if confirmed by future work, may have implications for future studies wishing to incorporate noise- and/or tone-vocoded speech stimuli as a way of increasing task difficulty while preserving reliably high levels of intelligibility and perception of talker characteristics. Tone-vocoded stimuli are sometimes used in studies of masking to preserve intelligibility while minimizing/varying the spectral overlap of competing sounds (e.g., Arbogast *et al.*, 2002, 2005) or to manipulate the presence/absence of spatial cues (Swaminathan *et al.*, 2016) or talker gender cues in masked listening experiments (Brungart *et al.*, 2006). By varying the number of vocoded channels for either type of carrier, intelligibility may be altered while other features such as pitch, intonation, or general quality of speech also change. Based on the current findings, there appear to be vocoded conditions in which intelligibility is at ceiling while gender classification performance is reduced, and strong biases in gender classification are apparent for noise vocoding. Finally, these results, and future studies of this topic, may further our understanding about how listeners perceive and classify talker gender, a topic that may become more complex as the cultural understanding of gender—and perhaps also the average listener's understanding of what it means to “sound male” or “sound female”—continues to evolve.

## References

[1] Arbogast, T. L., Mason, C. R., and Kidd, G., Jr. (2002). “ The effect of spatial separation on informational and energetic masking of speech,” J. Acoust. Soc. Am. 112, 2086–2098. 10.1121/1.1510141 12430820

[2] Arbogast, T. L., Mason, C. R., and Kidd, G., Jr.(2005). “ The effect of spatial separation on informational masking of speech in normal-hearing and hearing-impaired listeners,” J. Acoust. Soc. Am. 117(4), 2169–2180. 10.1121/1.1861598 15898658

[3] Brown, K. M., Dahl, K. L., Cler, G. J., and Stepp, C. E. (2021). “ Listener age and gender diversity: Effects on voice-based perception of gender,” J. Voice 35(5), 739–745. 10.1016/j.jvoice.2020.02.004 32165021 PMC7483284

[4] Brungart, D. S. (2001). “ Informational and energetic masking effects in the perception of two simultaneous talkers,” J. Acoust. Soc. Am. 109(3), 1101–1109. 10.1121/1.1345696 11303924

[5] Brungart, D. S., Iyer, N., and Simpson, B. D. (2006). “ Monaural speech segregation using synthetic speech signals,” J. Acoust. Soc. Am. 119(4), 2327–2333. 10.1121/1.2170030 16642846

[6] Dorman, M. F., Loizou, P. C., and Rainey, D. (1997). “ Speech intelligibility as a function of the number of channels of stimulation for signal processors using sine-wave and noise-band outputs,” J. Acoust. Soc. Am. 102(4), 2403–2411. 10.1121/1.419603 9348698

[7] Dudley, H. (1939). “ Remaking speech,” J. Acoust. Soc. Am. 11, 169–177. 10.1121/1.1916020

[8] Fu, Q. J., Chinchilla, S., and Galvin, J. J. (2004). “ The role of spectral and temporal cues in voice gender discrimination by normal-hearing listeners and cochlear implant users,” J. Assoc. Res. Otolaryngol. 5(3), 253–260. 10.1007/s10162-004-4046-1 15492884 PMC2504551

[9] Fu, Q. J., Chinchilla, S., Nogaki, G., and Galvin, J. J. III (2005). “ Voice gender identification by cochlear implant users: The role of spectral and temporal resolution,” J. Acoust. Soc. Am. 118(3), 1711–1718. 10.1121/1.1985024 16240829

[10] Fuller, C. D., Gaudrain, E., Clarke, J. N., Galvin, J. J., Fu, Q. J., Free, R. H., and Başkent, D. (2014). “ Gender categorization is abnormal in cochlear implant users,” J. Assoc. Res. Otolaryngol. 15(6), 1037–1048. 10.1007/s10162-014-0483-7 25172111 PMC4389960

[11] Gonzalez, J., and Oliver, J. C. (2005). “ Gender and speaker identification as a function of the number of channels in spectrally reduced speech,” J. Acoust. Soc. Am. 118(1), 461–470. 10.1121/1.1928892 16119365

[12] Kidd, G., Jr., Mason, C. R., Best, V., Roverud, E., Swaminathan, J., Jennings, T., Clayton, K., and Colburn, S. H. (2019). “ Determining the energetic and informational components of speech-on-speech masking in listeners with sensorineural hearing loss,” J. Acoust. Soc. Am. 145(1), 440–457. 10.1121/1.5087555 30710924 PMC6347574

[13] Kidd, G., Jr., Mason, C. R., Swaminathan, J., Roverud, E., Clayton, K. K., and Best, V. (2016). “ Determining the energetic and informational components of speech-on-speech masking,” J. Acoust. Soc. Am. 140(1), 132–144. 10.1121/1.4954748 27475139 PMC5392100

[14] Loizou, P. C., Dorman, M., and Tu, Z. (1999). “ On the number of channels needed to understand speech,” J. Acoust. Soc. Am. 106(4), 2097–2103. 10.1121/1.427954 10530032

[15] Middlebrooks, J. C., Simon, J. Z., Popper, A. N., and Fray, R. R. (2017). *The Auditory System at the Cocktail Party* ( Springer Nature, Basingstoke, UK).

[16] Roberts, B., Summers, R. J., and Bailey, P. J. (2011). “ The intelligibility of noise-vocoded speech: Spectral information available from across-channel comparison of amplitude envelopes,” Proc. R. Soc. B: Biol. Sci. 278(1711), 1595–1600. 10.1098/rspb.2010.1554 PMC3081737 21068039

[17] Schvartz, K. C., and Chatterjee, M. (2012). “ Gender identification in younger and older adults: Use of spectral and temporal cues in noise-vocoded speech,” Ear Hear. 33(3), 411–420. 10.1097/AUD.0b013e31823d78dc 22237163 PMC3340495

[18] Scott, S. K., Rosen, S., Lang, H., and Wise, R. J. (2006). “ Neural correlates of intelligibility in speech investigated with noise vocoded speech—A positron emission tomography study,” J. Acoust. Soc. Am. 120(2), 1075–1083. 10.1121/1.2216725 16938993

[19] Shannon, R. V., Zeng, F. G., Kamath, V., Wygonski, J., and Ekelid, M. (1995). “ Speech recognition with primarily temporal cues,” Science 270(5234), 303–304. 10.1126/science.270.5234.303 7569981

[20] Smith, Z. M., Delgutte, B., and Oxenham, A. J. (2002). “ Chimaeric sounds reveal dichotomies in auditory perception,” Nature 416(6876), 87–90. 10.1038/416087a 11882898 PMC2268248

[21] Souza, P., and Rosen, S. (2009). “ Effects of envelope bandwidth on the intelligibility of sine- and noise-vocoded speech,” J. Acoust. Soc. Am. 126(2), 729–805. 10.1121/1.3158835 PMC2730710 19640044

[22] Studebaker, G. A., Pavlovic, C. V., and Sherbecoe, R. L. (1987). “ A frequency importance function for continuous discourse,” J. Acoust. Soc. Am. 81(4), 1130–1138. 10.1121/1.394633 3571730

[23] Swaminathan, J., Mason, C. R., Streeter, T., Best, V., Roverud, E., and Kidd, G., Jr. (2016). “ Role of binaural temporal fine structure and envelope cues in cocktail-party listening,” J. Neurosci. 36(31), 8250–8257. 10.1523/JNEUROSCI.4421-15.2016 27488643 PMC4971368

[24] Whitmal, N. A. III, Poissant, S. F., Freyman, R. L., and Helfer, K. S. (2007). “ Speech intelligibility in cochlear implant simulations: Effects of carrier type, interfering noise, and subject experience,” J. Acoust. Soc. Am. 122(4), 2376–2388. 10.1121/1.2773993 17902872

[25] Willis, S., Xu, K., Thomas, M., Gopen, Q., Ishiyama, A., Galvin, J. J. III, and Fu, Q. J. (2021). “ Bilateral and bimodal cochlear implant listeners can segregate competing speech using talker sex cues, but not spatial cues,” JASA Express Lett. 1(1), 014401. 10.1121/10.0003049 33521793 PMC7814501

[26] Xu, L., Xi, X., Patton, A., Wang, X., Qi, B., and Johnson, L. (2021). “ A cross-language comparison of sentence recognition using American English and Mandarin Chinese HINT and AzBio sentences,” Ear Hear. 42(2), 405–413. 10.1097/AUD.0000000000000938 32826510

[27] Yun, D., Jennings, T. R., Kidd, G., Jr., and Goupell, M. J. (2021). “ Benefits of triple acoustic beamforming during speech-on-speech masking and sound localization for bilateral cochlear-implant users,” J. Acoust. Soc. Am. 149(5), 3052–3072. 10.1121/10.0003933 34241104 PMC8102069

